# Local Marketing of a National Texting-Based Smoking Cessation Program: Is It Cost Effective?

**DOI:** 10.3389/fpubh.2020.00116

**Published:** 2020-05-07

**Authors:** Henry Shelton Brown, Ujas Patel, Sarah Seidel, Ashley LeMaistre, Kim Wilson

**Affiliations:** ^1^UTHealth, University of Texas School of Public Health, Austin, TX, United States; ^2^Austin Public Health, Austin, TX, United States

**Keywords:** smoking cessation, cost-effectiveness, texting, social marketing, tobacco, cigarettes

## Abstract

Tailored texting interventions for smoking cessation are increasingly popular given the ubiquitousness of smart phones. Because high development costs and limited expertise may pose substantial barriers to designing and implementing these programs at the local level, utilization of existing programs at the national level is a promising strategy. In 2011, Austin Public Health focused on promoting smoking cessation among Austin/Travis County residents. Their strategy involved marketing and linking their citizens to a federally-funded, evidence-based smoking cessation program via texting. The target audience was low income, 18–24 year olds. Their marketing strategies included radio ads, digital ads, social media ads, and direct outreach at events in Austin, Texas. During the period between April 2016 and July 2017, 1,022 people signed up for the program. The quit rate was comparable to other texting programs which were tailored at the local level, and the program was cost-effective, costing $12,704.56 per life-year added, averting $99.38 per person in medical costs, discounted at 3%.

## 1. Introduction

As technological improvements in smart phones have accelerated, tailored texting interventions for smoking cessation have become more popular (1). A key advantage of text-based interventions is the near universal usage of smart phones, which can be used anywhere. Almost anyone can be enrolled, and can be set up to receive the program in a matter of minutes. Several tailored texting programs have been created, and two have been found to be cost-effective (1, 2).

In cost-effectiveness analysis, program development is not included in costs. This is because development costs would not have to be replicated if the program were continued. The cost of extending the program is what is relevant in cost effectiveness (3). At the margin, the additional cost of adding a person to texting programs can be low, even though development is typically ongoing in reality. Algorithms are written, and so the “tailoring” is automatic. However, the reality is that programs need funding to operate, and previously developed tailored algorithms are rarely shared. Therefore, efficient scale economies are rarely achieved. In addition to excessive costs due to duplication of development, local expertise is likely to vary in both the quality of the text messaging, the technical ability to program the algorithms, the hosting of the program on the server, etc. Development costs and expertise limits likely affect the reach and the effectiveness of many programs.

In contrast to other programs, Austin Public Health chose to utilize an existing federal program, the National Cancer Institute (NCI)'s SmokefreeTXT. This free federal program was created to provide adults (and young adults, specifically) with 24/7 support for smoking cessation, including encouraging messages, advice, and tips to help smokers quit smoking and stay quit. Using the existing text-based development and operation, and thereby not having to design and implement an individualized texting program allowed the Austin team to devote their energies to marketing the program. They placed ads on the radio, the internet, and in social media. They also direct marketed at various events around town. The purpose of this report is to assess this program from the perspective of cost-effectiveness analysis.

## 2. Background

Given the high costs and poor health outcomes for smokers, smoking cessation programs generally have been found to be cost-effective (4, 5). Smoking cessation programs are cost-effective among school children (6), as are smoking prevention programs among youth (7).

There are a few cost effectiveness studies of smoking cessation programs which were text- or computer-based. In a randomized controlled trial in the UK, Wu et al. (8) evaluated a computer tailored smoking cessation intervention and found it slightly more cost effective than a generic, non-tailored “self-help” smoking cessation intervention. Stanczyk et al. (1) compared text and video-based smoking cessation interventions in a randomized controlled trial in the Netherlands and found mixed results. However, both intervention types were cost-effective. Guerriero et al. (2) evaluated a text-based cessation intervention much like SmokefreeTXT and found it to be cost-effective. Finally, Xu et al. (9) evaluated a national mass media campaign aimed at all smokers nationwide and also found it to be cost-effective. However, none of these programs were targeted at low-income adults.

### 2.1. NCI's SmokefreeTXT

SmokeFreeTXT is a free program from the National Cancer Institute designed for smokers ages 13 and up and lasting 6–8 weeks (https://smokefree.gov/tools-tips/text-programs). When an individual signs up, they are asked to enter a desired quit date. NCI begins sending text messages 2 weeks before the quit date and continues to send messages for 6 weeks after it. Users receive from one to five messages a day, approximately 130 messages in total. NCI continues to send follow-up texts 1, 3, and 6 months after the program ends. Automated messages include clinical content, assessments, and user-initiated feedback.

Clinical content messages provide information on the physical and behavioral effects of smoking while providing strategies for successful quitting. Assessment messages are used to gauge user's mood, craving level, and smoking status. For example, a message will ask a user to describe their craving level as “high, medium, or low.” Based on the response, SmokefreeTXT will send a tailored message with words of encouragement. In addition to the scheduled messages, users can prompt the system at any time for support by texting the keywords “crave,” “mood,” and “slip.” The system then sends messages of positive reinforcement, such as “So you slipped. That doesn't mean you failed. Take this as an opportunity to learn how to avoid lighting up next time. You will be happy you did.”

Note that the Austin marketing program drove enrollees to the NCI program, but that we did not expect an additional, individual quitting effect for the Austin component.

## 3. Materials and Methods

The main benefit of SmokefreeTXT was life-years added. We decided not to adjust for quality of life. Stapleton points out that the appropriate weighting of life-years added of former smokers is controversial (10). For example, some researchers weight life-years added of former smokers at 0.9, while others weight them at over 1 because cessation not only adds life-years, but it reduces morbidity. For most years of a smoker's life, measures of activities of daily living don't vary by smoking status. For instance, smokers can shop, which is an activity of daily living, for themselves for years, but then may need help at the onset of a smoking-related illness at the end of life. For these reasons, we conservatively weighted life-years at 1, again following Stapleton.

We took a societal perspective on costs. That is, we included any cost by any entity, including unpaid opportunity costs by Austin Public Health or enrollees. However, this intervention had no volunteers, and enrollee opportunity costs were nil. The majority of the costs were the marketing SmokefreeTXT.

We applied a discount rate of 3% to all cost and benefits in the study. All analyses were performed with the R package Health Economic Evaluation (heemod) (11).

The benefits were life-years added. The averted medical costs were the medical costs saved by not smoking by age. The method for estimating averted medical costs and life years added are described below. The program costs were those of SmokefreeTXT, as described below in **Table 2**.

### 3.1. The Markov Chain

In order to estimate benefits, one must be able to estimate life-years added by the program. Markov chain models are the standard method for estimating life-years added. The idea is to compare the life-years obtained, hypothetically, for a population of smokers with and without SmokefreeTXT. If the additional life-years are greater with SmokefreeTXT, those are part of the benefits of the program.

**Figure 1:**
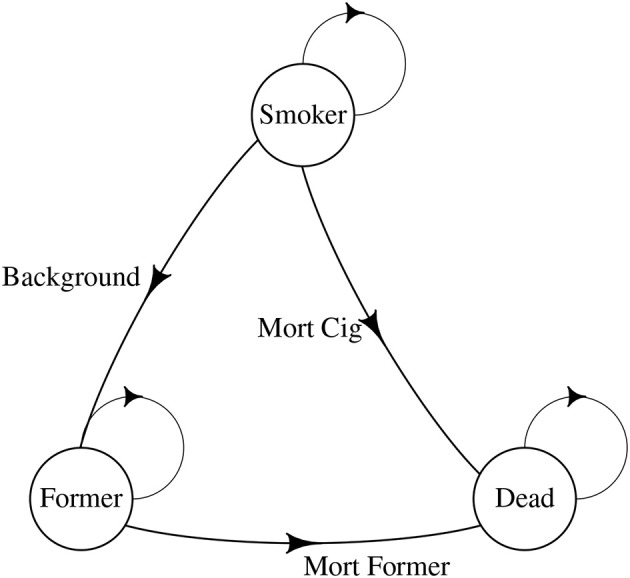
Tobacco Markov chain.

**Figure 2:**
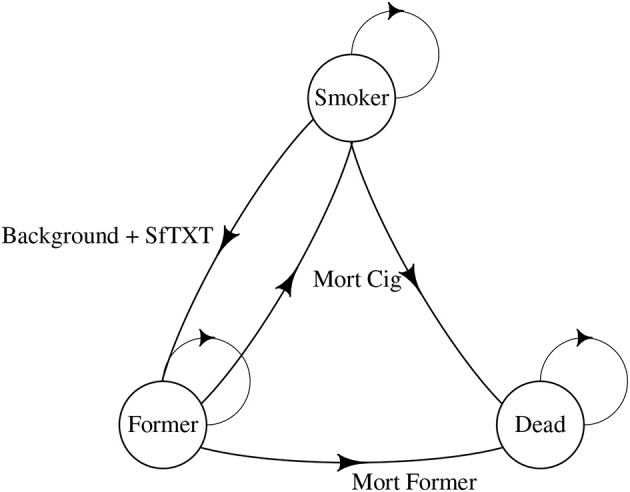
SmokefreeTXT's Markov chain.

Figures 1, 2 illustrate our approach. Our model started with 1,000 smokers. As with all Markov Chains, people move from one state to another, with probabilities being determined by the person's previous state. In our model, the states were remained smokers, became former smokers, or died. There was a 2% background quit rate per year, meaning up to 98% of smokers remained smokers from year-to-year with no intervention, depending on the mortality rate. The quit rate for those in the program was empirically derived from the program and is illustrated in Figure 2, as were costs (see below). We assumed a 30% relapse rate for quitters in the year after quitting. We started our age at 20 and continued to age 80.

### 3.2. Mortality

In our Markov Chain, mortality rates varied by age. Following Guerriero et al. (2), Table 1 gives the mortality by age. Note that these parameters are for British smokers, but the mortality rates should be the same for our population.

**Table 1:** Mortality rates by age.

**Age**	**Smoker**	**Former smoker**
20–44	0.00280	0.0020
45–54	0.00810	0.00490
55–64	0.02030	0.01340
65–74	0.04700	0.03160
75–84	0.10600	0.07730
85+	0.21870	0.17970

### 3.3. The Monte Carlo

Finally, we conducted a Monte Carlo sensitivity analysis to test the sensitivity of our analysis to changes in the cost of the program, the effect size, the background quit rate, and the relapse rate.

### 3.4. Cost of Smoking by Age

**Figure 3:**
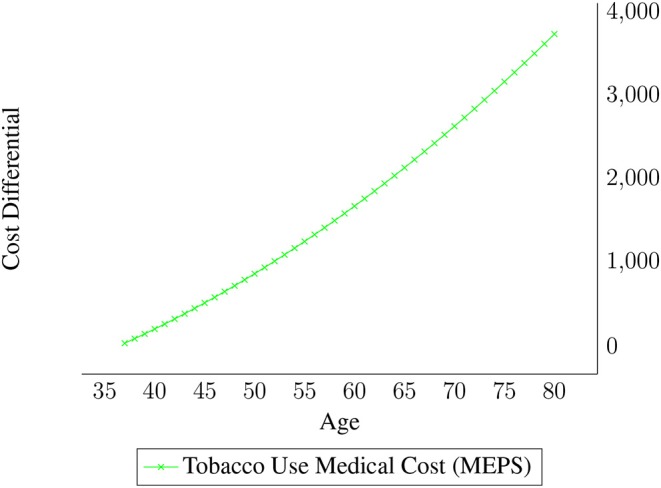
Differential medical costs by age for smokers.

Our Markov chain also allowed costs of smoking to vary by age. Figure 3 displays the average yearly tobacco health expense differential for smokers by age. The estimates are from the 2012 Medical Expenditure Panel Survey, and focus on the medical cost differentials between smokers and non-smokers (12). The cost estimates in Figure 3 are from a regression analysis performed by UTSPH, where monthly medical cost differentials were regressed on age and age squared. Average costs were lower for smokers than non-smokers under 37, but the lower medical costs for that age group likely reflect smokers' attitudes about health rather than cost savings due to smoking. Therefore, those cost differentials were set to zero for the ages 33–37 in the Markov Chain.

## 4. Results

### 4.1. Intervention Benefits

**Figure 4:**
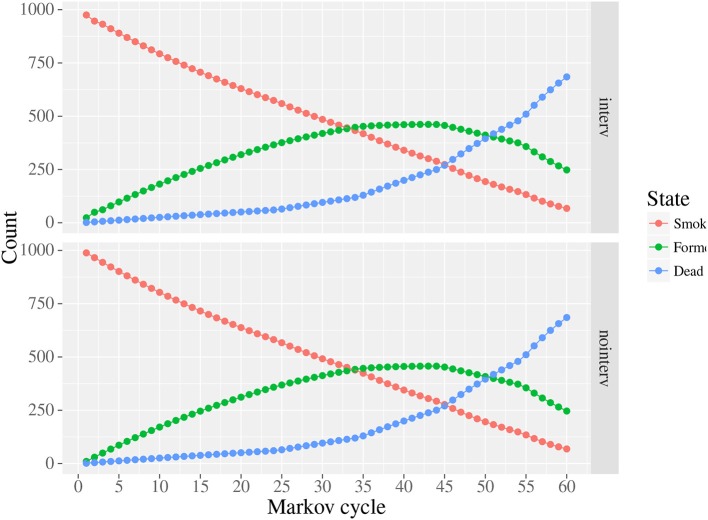
Markov chain life-years by cycle.

Overall, there were 9.5 additional life-years per 1,000 smokers in the intervention versus no intervention. The life-years were discounted at 3%. Note that the actual undiscounted number of life-years added was 4 times higher. Figure 4 plots the states over time with and without the intervention. Time zero refers to age 20, and time 60 refers to age 80. All of the movement, or reduction in smoking, is at ages 20 and 21, and there is a slight dip in the growth of former smokers at age 21 due to the relapse.

### 4.2. Intervention Costs

Table 2 lists the costs of the intervention, broken down by staffing or personnel costs, and the costs of smoking cessation outreach marketing. The APH staff contribution to the SmokefreeTXT intervention was lean because APH staff primarily managed the promotion and marketing of SmokefreeTXT rather than developing the texting program itself, as noted earlier. Marketing strategies included radio ads, digital ads, social media ads, and direct outreach at events in Austin, Texas. Table 2 reveals that most of the money was spent on the marketing in an iterative, trial and error manner.

**Table 2:** SmokefreeTXT program cost summary.

**Period: May 2016–April 2017**		
**Staffing**		
Program coordinator	$16,013	
Principal investigator	$2,853	
Data analyst	$4,249	
NCI staff	$4,500	
Media consultants and buys		“Reach/# Impressions”
Work plan development	$15,328	
Digital	$13,250	1,155,117
Radio	$48,000	9,455,210
Social	$16,500	1,317,701
Events	$65,000	38,875
Street teams	$35,525	860
Supplies		
Incentives/Giveaways	$3,140	
Total program costs	$224,358	11,967,763

*Calculations based on data provided by Austin Public Health*.

### 4.3. Averted Medical Costs

Our Markov Chain results show that the intervention led to $99.38 of averted medical costs per enrollee, discounted at 3%. Note that the averted costs were much higher, but as shown in Figure 3, the cost differentials increase exponentially later in life, but are then discounted at a higher rate.

### 4.4. Cost Effectiveness Results

SmokefreeTXT added 9.5 life-years per 1,000 smokers enrolled, costing $12,704.56 per life-year added. This is in line with, but higher, than published results for the most similar program identified [see e.g., (2)].

### 4.5. Sensitivity Analysis

**Figure 5:**
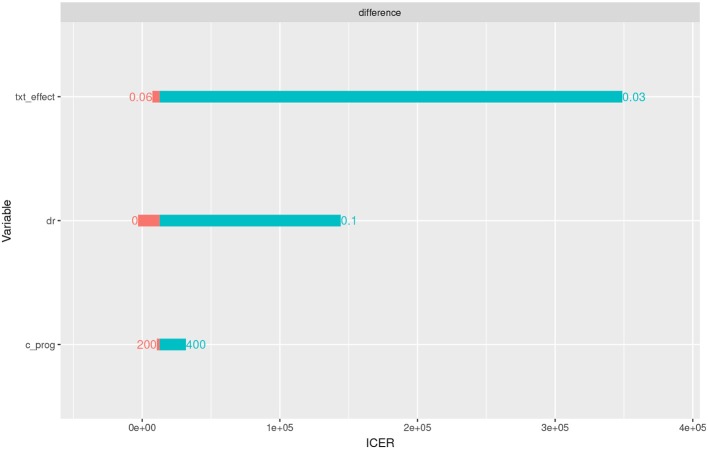
One-way sensitivity analysis.

We conducted three one-way sensitivity analyses, as illustrated in Figure 5. First, the program effect (txt_effect) was varied from 0.03 (0.01 effect size plus 0.02 background) to 0.06. If the program effect size was only 0.01, the program was not cost-effective. The discount rate (dr) was varied from 0 to 0.1, but the cost effectiveness result stands under 0.05. Finally, the cost per enrollee was varied from $200 to $400, but the program was still cost-effective.

**Figure 6:**
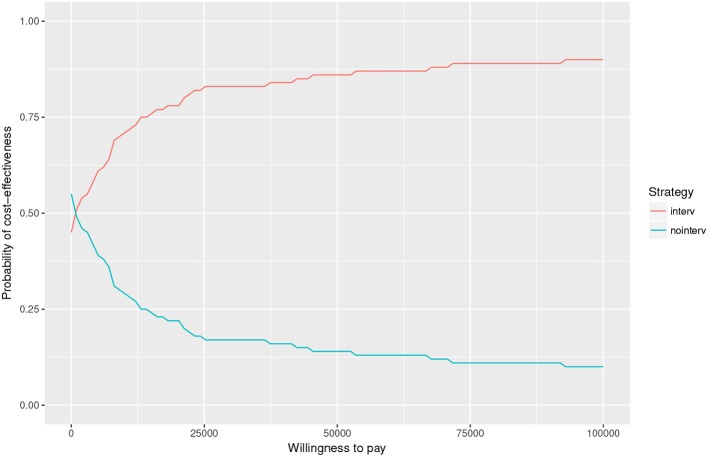
Monte Carlo sensitivity analysis.

We also conducted a Monte Carlo analysis (see Figure 6). We modeled the effect size of SmokefreeTXT as a Beta distribution, the cost of the program as a Gamma distribution, the background quit rate as a Beta, and the relapse rate as a Beta. We repeated the draws 100 times. At a low willingness to pay value of $25,000 per life-year added, the chance of cost-effectiveness was over 99.38%.

Note that we paid special attention to the cost per enrollee. This is due to not knowing the cost of programming per enrollee from NCI.

## 5. Discussion

SmokefreeTXT combined local social marketing with a federal smoking cessation texting intervention. Our results showed that 1,022 people signed up, and roughly 5.28% people quit (reporting that they had at least quit for at least 3 months upon last correspondence), adding 9.5 discounted life-years per 1,000. The quit rate was comparable to other texting programs which were tailored at the local level, and the program was cost-effective, costing $12,704.56 per life-year added.

The fact that the national program SmokefreeTXT was able to offer a competitive program in terms of effectiveness and cost-benefit analysis in a local market is positive for other entities seeking to lower smoking prevalence in a cost-efficient manner. Small entities with little expertise in text messaging do not need to incur the expense of creating another texting intervention. They can simply locally market the text messaging service available at the national level. This will lower costs due to scale economies, and improve effectiveness due to increased expertise.

More generally, technological improvements such as smart phones afford the possibility of partnerships to form between local communities and higher levels of government. Programs can be developed and shared with local governments and local health departments, taking advantage of expertise and scale economies.

Our Monte Carlo model revealed that local marketing of SmokefreeTXTs was robust to variations in parameters in terms of cost-effectiveness. In the one-way sensitivity analysis, the results were sensitive to effect sizes.

There were several limitation to our analysis. First, we do not have demographic data on participants. Second, we did not include NCI's programming costs because we do not have an estimate. However, we believe the cost per enrollee must be low given that this program is used nationally.

## Data Availability Statement

The datasets for this study will not be made publicly available because the authors do not own the data. They were shared by the National Cancer Institute. Requests to access these datasets should be directed to HB, Henry.S.Brown@uth.tmc.edu.

## Ethics Statement

The study was submitted to UTHealth's IRB committee and was determined to be exempt.

## Author Contributions

HB led the team, wrote the first draft, and performed the analysis. SS and AL wrote sections of the paper and helped us analyze the National Cancer Institute data as well as cost data. KW advised on the methods and wrote and edited several rounds, as did UP.

## Conflict of Interest

The authors declare that the research was conducted in the absence of any commercial or financial relationships that could be construed as a potential conflict of interest.
